# Oil-Based Double Emulsion Microcarriers for Enhanced Stability and Bioaccessibility of Betalains and Phenolic Compounds from *Opuntia stricta* var. *dillenii* Green Extracts

**DOI:** 10.3390/foods12112243

**Published:** 2023-06-01

**Authors:** Sara Parralejo-Sanz, Iván Gómez-López, Erika González-Álvarez, Mara Montiel-Sánchez, M. Pilar Cano

**Affiliations:** 1 Laboratory of Phytochemistry and Functionality of Plant Foods, Department of Biotechnology and Food Microbiology, Institute of Food Research (CIAL) (CSIC-UAM), 28049 Madrid, Spain; sara.parralejo@csic.es (S.P.-S.); ivan.gomez@ehu.eus (I.G.-L.); akire_goa@yahoo.com.mx (E.G.-Á.); mara.montiels@hotmail.com (M.M.-S.); 2 Nutrition and Obesity Group, Department of Nutrition and Food Science, Faculty of Pharmacy and Lucio Lascaray Research Center, University of the Basque Country (UPV/EHU), 01006 Vitoria-Gasteiz, Spain; 3 Unidad de Investigación y Desarrollo en Alimentos, TecNM/Instituto Tecnológico de Veracruz, Miguel Ángel de Quevedo 2779, Veracruz 91897, Mexico

**Keywords:** *Opuntia stricta* var. *dillenii* fruits, betalin and phenolic green extracts, double emulsions, in vitro gastro-intestinal digestion, microscopic studies, bioactive bioaccessibility

## Abstract

Opuntia cactus fruit (prickly pear flesh and agricultural residues such as peels and stalks) is an important source of bioactive compounds, including betalains and phenolic compounds. In this work, two double emulsion W_1_/O/W_2_ formulations (A and B) were designed to encapsulate green extracts rich in betalains and phenolic compounds obtained from *Opuntia stricta* var. *dillenii* (*OPD*) fruits with the aim of improving their stability and protecting them during the in vitro gastrointestinal digestion process. In addition, the characterization of the double emulsions was studied by microscopy and the evaluation of their physical and physico-chemical parameters. Formulation A, based on Tween 20, showed smaller droplets (1.75 µm) and a higher physical stability than Formulation B, which was achieved with sodium caseinate (29.03 µm). Regarding the encapsulation efficiency of the individual bioactives, betalains showed the highest values (73.7 ± 6.7 to 96.9 ± 3.3%), followed by flavonoids (68.2 ± 5.9 to 95.9 ± 7.7%) and piscidic acid (71 ± 1.3 to 70.2 ± 5.7%) depending on the formulation and the bioactive compound. In vitro digestive stability and bioaccessibility of the individual bioactives increased when extracts were encapsulated for both formulations (67.1 to 253.1%) in comparison with the non-encapsulated ones (30.1 to 64.3%), except for neobetanin. Both formulations could be considered as appropriate microcarrier systems for green *OPD* extracts, especially formulation A. Further studies need to be conducted about the incorporation of these formulations to develop healthier foods.

## 1. Introduction

**Figure 1:**
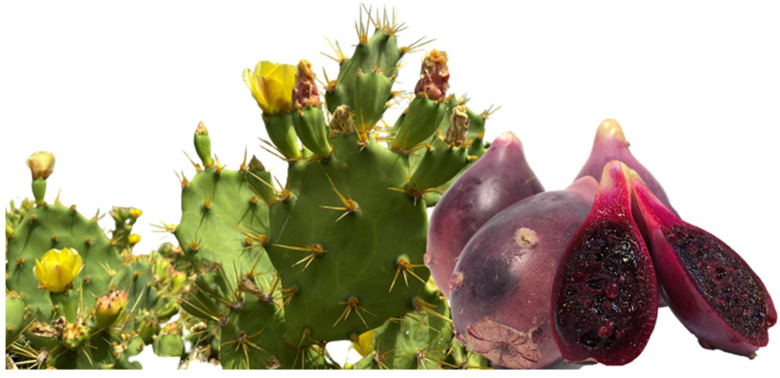
Image of *Opuntia stricta* var. *dillenii* cactus and whole fruit from the Canary Islands (Spain).

Prickly pear (*Opuntia* spp.) is the fruit of the dicotyledonous angiosperm plant called nopal cactus which belongs to the botanical family *Cactaceae*. It can be found in various arid and semi-arid areas around the world, with Mexico being the main producer. It is also cultivated in abundance in Italy, South Africa, Chile, Argentina and Spain, among others [1]. In Spain, prickly pear’s production is widespread throughout the Canary Islands, and it is commonly consumed as a fresh fruit. Plantations of this genus achieve easy expansion in clonal colonies due to their low water requirements and the climatic conditions associated to the Mediterranean Sea area [2]. To date, more than 200 species of *Opuntia* spp. have been identified [3]. The most commercialised and consumed species is *Opuntia ficus-indica*, but there are few others growing wildly, such as *Opuntia stricta* var. *dillenii* (OPD) (Figure 1), which is the subject of the present study. This wild variety is less investigated, and recent studies declared the need for further research due to its favorable nutritional composition, which may be of great interest to the food and pharmaceutical industries [4].

OPD’s fruits are a rich source of bioactive compounds such as betalains (responsible for their purple-coloured peel and pulp), especially betanin, isobetanin and neobetanin; phenolic acids, mainly piscidic acid, and flavonoids, principally quercetin and isorhamnetin glycosides [5]. These families of compounds have been recently studied and demonstrated potential in vitro antioxidant, anti-inflammatory, hypoglycaemic, immunomodulatory and anti-obesity properties, among others [6,7,8]. Some investigators have also studied benefits attributable to betalains and phenolic compounds from *Opuntia ficus-indica* in cell cultures, animal models and clinical trials [9,10]. 

Despite the biological potential of betalains and phenolic compounds, their chemical stability is conditioned by various intrinsic and extrinsic factors that drive their degradation, such as high temperatures, light exposure, O_2_, a low degree of glycosylation or acylation, degradation enzymes, pH values < 3 or >7, etc. [11,12]. From a physiological point of view, the degradation of betalains occurs during gastro-intestinal digestion in the stomach and the small intestine, leading to a decrease in their absorption and bioaccessibility. Meanwhile, phenolic compounds’ bioaccessibility is affected by their association with other molecules present on the food matrix such as polysaccharides or fats [5,13].

In order to enhance the stability and bioaccessibility of bioactive compounds, the strategies of choice at present are encapsulation by double emulsion, spray drying using different polymers, ionic gelation and hydrogels, among others [14]. Encapsulation with polysaccharides is also an interesting technique to improve the bioaccessibility of OPD bioactives. Some authors reported that the microencapsulation with maltodextrin and microcrystalline cellulose was considered a good formulation to prolong the stability of the bioactive compounds of the extracts of prickly pear during digestion and to increase their bioaccessibility, i.e., around 91.8% for betanin [13].

The technique chosen for the present research was the encapsulation of OPD green extracts by double emulsion microcarrier systems obtained by ultrasounds, specifically water-in-oil-in-water (W_1_/O/W_2_) emulsions, as they are the most suitable for the protection of hydrophilic compounds, such as betalains and phenolic compounds. Double emulsion multi-interface structure endows them with a peculiar sequential digestion characteristic, in which their components are released along the digestive sequence from the external aqueous interface to the oil phase and finally from the internal aqueous phase. This property makes it a promising strategy as the dual emulsion acts as a vehicle for programmed and protected release of labile ingredients [15]. In addition, it is credited with improved storage stability due to a reduced interaction between the components of the food matrix, and of their exposure to environmental factors [16].

The aim of the present research was to evaluate the impact of encapsulation by two different double emulsion microcarrier water-in-oil-in-water systems (W_1_/O/W_2_) on the digestive stability and bioaccessibility of the main individual betalains and phenolic compounds present in green extracts from *Opuntia stricta* var. *dillenii’s* whole fruits. In addition, the characterization of the double emulsions was studied by optical and confocal microscopy, conducting the evaluation of their physical and physico-chemical parameters during cold storage in order to stablish their stability. 

## 2. Materials and Methods

### 2.1. Chemicals

Ultrapure MiliQ water was obtained from a Millipak^®^ Express 40 system Merk-Milipore (Darmstadt, Germany). Ethanol (99.97%) was obtained from VWR International (Barcelona, Spain). Betanin was extracted, isolated and purified from lyophylized beetroots (*Beta vulgaris* subsp. *vulgaris*) using a Sephadex LH-20 resin purchased from Sigma-Aldrich (St. Louis, MO, USA). Piscidic acid was purified from prickly pear peels by semi-preparative high-performance liquid chromatography (HPLC) by the protocol also described by García-Cayuela et al. (2019) [17]. The standards of isorhamnetin glycosides were supplied from Dr. Serna-Saldivar from the Biotechnology center FEMSA (Escuela de Ingeniería y Ciencias, Instituto Tecnológico de Monterrey, Monterrey, Mexico).

For the double emulsion (W_1_/O/W_2_) formulations, medium-chain triglycerides (MCT) from coconut oil were purchased from Ketosource Ltd. (London, UK). NaCl was obtained from Panreac Química S.L.U. (Barcelona, Spain). Glycerol, sodium caseinate salt and Tween 20% were purchased from Sigma-Aldrich (St. Louis, MO, USA). Merck KGaA (Darmstadt, Germany) supplied Buffer TRIS pH 9 (Tris (hydroxymethyl)aminomethane). Gelatin and Arabic gum were obtained from Acros Organics (Geel, Belgium) and guar gum was obtained from MCS (Puebla, Mexico). Polyglycerol polyricinoleate (PGPR) and phosphatidylcholine (PC) were purchased from Palsgaard A/S (Juelsminde, Denmark) and Avanti Polar Lipids (Alabaster, AL, USA), respectively. Chloroform was obtained from Thermo Fisher Technologies (Waltham, MA, USA). Lipase enzyme (RGE15) was purchased from Lipolytech (Marseille, France). Amylase (10080), pepsin (P6887), pancreatin (P7545), bile salts (B838) and other reagents used for the in vitro digestion assay were supplied by Sigma-Aldrich (St. Louis, MO, USA).

### 2.2. Plant Material

*Opuntia stricta* var. *dillenii* (OPD) fruits were harvested in September of 2020 in Tenerife, Canary Islands, Spain (29°3′ N, 13°4′ W; 209 m above sea level), Figure 1. The fruits were selected according to the size, colour, and maturity; the damaged ones were rejected. The maturity was selected from previous studies conducted with this Opuntia fruit when the full colour of the fruit pulp developed [5]. Analysis of the physico-chemical parameters of fresh fruits was conducted; data are shown in Table S1 (Supplementary Materials). The stabilization of the OPD tissues was carried out by freezing by liquid nitrogen, followed by tissue freeze-drying according to Gómez-López et al. (2021a) [5] and stored at −24° until their use to obtain the OPD green extracts.

### 2.3. Obtaining OPD’s Betalain- and Phenolic-Rich Green Extracts 

The extraction of betalains and phenolic compounds from lyophilized OPD whole fruits was performed by ultrasound-assisted extraction (UAE), using ethanol:water (15:85, *v*/*v*) as green solvent, as reported by Gómez-López et al. (2021b) [18]. The ultrasound equipment was a Digital sonifier (Branson Ultrasonics Corporation, Danbury, CT, USA) with a 13 mm diameter ultrasound probe (Biogen Cientific S.L, Madrid, Spain).

### 2.4. Characterization of Betalains and Phenolic Compounds on OPD Green Extracts

Green OPD extracts were dissolved in ultrapure water and filtered by 0.45 µm nylon filters (Análisis Vínicos, Ciudad Real, Spain) for HPLC analysis. Individual betalains and phenolic compounds were simultaneously determined according to the method reported by Gómez-López et al. (2021a) [5].

Briefly, 1200 Series Agilent HPLC System (Agilent Technologies, Barcelona, Spain) with a C18, reverse column Zorbax SB-C18, 250 × 4.6 nm i.d., S-5 μm (Agilent Technologies, Santa Clara, CA, USA) at 25 °C was employed. Ultrapure water with 1% formic acid (*v*/*v*) (Phase A) and methanol (99.8% LC-MS) with 1% formic acid (*v*/*v*) (Phase B) were used in gradient for 70 min in order to obtain the optimal separation of bioactive compounds. A flow rate of 0.8 mL/min and an injection volume of 20 μL were used. The UV–Visible photodiode array detector was set at four wavelengths: 280 nm for phenolic acids, 370 nm for flavonoids, 480 nm for betaxanthins and 535 nm for betacyanins. To confirm the chemical composition of each bioactive compound, HPLC-DAD-MS/QTOF and HPLC-DAD-ESI/MS analysis was also conducted. Gómez-López et al. (2021a) [5] reported the complete description of the UV–vis and mass spectroscopy characteristics of all individual betalains and phenolic compounds found in *OPD* green extracts. 

### 2.5. Formation of Double Emulsion Systems W_1_/O/W_2_

Two different formulations, Formulation A and Formulation B, were elaborated to encapsulate OPD whole fruit green extracts. Both double emulsion systems (W_1_/O/W_2_) were prepared following two steps: (1) Inner water–oil emulsion (W_1_/O), where W_1_ was the aqueous solution, in which the OPD extract was dissolved and dispersed in the oil phase and (2) water-in-oil-in-water emulsion (W_1_/O/W_2_) emulsion system, in which the W_1_/O emulsion was dispersed in the secondary water phase, W_2_. Table 1 shows the composition of the two double emulsions used to encapsulate *Opuntia stricta* var. *dillenii* green extracts.

#### 2.5.1. Preparation of Formulation A

Formulation A was prepared following the method reported by Velderrain-Rodríguez et al. (2019) [19] and Kaimainen et al. (2015) [20] with modifications. Table 1 shows the complete composition of the assayed formulations A and B. In Formulation A, the inner water-in-oil emulsion (W_1_/O) was composed of 75% (*w*/*w*) of the oil phase (O) (70% (*w*/*w*) MCT oil and 5% (*w*/*w*) polyglycerol polyricinoleate PGPR) and 25% (*w*/*w*) of the aqueous phase (W_1_) (22% (*w*/*w*) 0.1 M NaCl solution containing the OPD betalain- and phenolic-rich green extract (750 mg) and 3% glycerol as co-surfactant). W_1_ dispersion into the oil phase (O) was performed by using an ultra-speed homogenizer, Ultra-Turrax, T-25 Digital homogenizer (IKA Wworks Inc., Staufen im Breisgau, Germany) at 7200 rpm for 10 min (4:2:4 min, ON:OFF:ON) with an external cooling using an ice-water bath, and continuously using an ultrasound equipment (Branson Ultrasonics Corporation, Brookfield, CT, USA) with a 13 mm diameter ultrasound probe (Biogen Cientifica S.L., Madrid, Spain). It worked at a 20 kHz frequency and a 70% amplitude for 3 min (1:1 sg, ON:OFF) also with external cooling by ice-water bath.

Double emulsion (W_1_/O/W_2_) was prepared with 25% (*w*/*w*) of the oil phase (the previous inner emulsion, W_1_/O) and 75% (*w*/*w*) of the secondary aqueous phase (W_2_) is composed by 73.5% (*w*/*w*) of a 0.1 M NaCl solution and a 1.5% (*w*/*w*) Tween 20, as a hydrophilic surfactant. The dispersion of W_1_/O into W2 was achieved using the Ultra-Turrax T-25 homogenizer at 3600 rpm for 5 min (2:1:2, min, ON:OFF:ON); after, the ultrasound equipment with a 13 mm diameter ultrasound probe and a 20 kHz frequency was used, running at a 60% amplitude during 90 s in continuous pulse mode with an external cooling with an ice-water bath. The obtained double emulsions were stored at −80 °C until HPLC analysis and at 7 °C for stability studies. In vitro digestion studies were conducted using freshly made formulation A (W_1_/O/W_2_ double emulsion).

#### 2.5.2. Preparation of Formulation B

Formulation B was composed of a primary W_1_/O emulsion which had a 70% (*w*/*w*) oil phase (O): (52% (*w*/*w*) MCT oil, 14% (*w*/*w*) polyglycerol polyricinoleate (PGPR), 4% (*w*/*w*) Phosphatidylcholine (PC) and 30% (*w*/*w*) aqueous phase (W_1_), in which OPD’s green extract (750 mg) was dissolved in 6% of gelatine in the TRIS buffer solution (pH 9), Table 1. For W1/O preparation, the oil phase (O) was homogenized during 1 min using an Ultra-Turrax, T-25 homogenizer at 4000 rpm. After that, W_1_ was dispersed into O by using the Ultra-Turrax, T-25 homogenizer again at 16,000 rpm, 3 cycles of 1 min:1 min (ON:OFF) with an external ice-water bath. In the second step, the ultrasound equipment with a 13 mm diameter ultrasound probe and 20 kHz frequency was used, running at a 30% amplitude during 2 cycles of 30 s (1 s:1 s, ON:OFF). also with an external ice-water bath.

Double emulsion (W_1_/O/W_2_) was prepared according to Casanova and Cardona (2009) [21] with modifications. It consisted of 30% (*w*/*w*) of W_1_/O and 70% (*w*/*w*) of the secondary aqueous phase (W_2_), made up of 56.8% (*w*/*w*) of a protein emulsifier, sodium caseinate at 3% (*w*/*w*), 13% (*w*/*w*) of NaCl 10% on glycerol, 0.265% (*w*/*w*) Arabic gum and 0.175% guar gum. Before the formation of the double emulsion, the secondary water phase was homogenized for 1 min using an Ultra-Turrax, T-25 homogenizer at 4000 rpm. The double emulsion (W_1_/O/W_2_) was obtained by applying the Ultra-Turrax, T-25 homogenizer at 11,000 rpm during 2 min in a continuous pulse mode. The obtained formulation was stored at −80 °C until bioactive HPLC analysis and at 7 °C for stability studies. In vitro digestion studies were conducted using freshly made Formulation B.

### 2.6. In Vitro Gastro-Intestinal Digestion 

The in vitro gastro-intestinal digestion assay was performed according to the standardised INFOGEST^®^ protocol [22] using green OPD extracts (as control) and 1.35 g of both double emulsion (W_1_/O/W_2_) system formulations (A and B) with a 19 mg *OPD* extract/g emulsion for Formulation A and a 22.5 mg OPD extract/g emulsion for Formulation B. The addition of the enzymes into the preparation of digestive fluids (saliva, gastric and duodenal) was performed daily prior to the digestion assays. Table S2 (Supplementary Materials) shows the composition of different phases of the in vitro gastro-intestinal assays. Theses assays were prepared twice for each sample (duplicate) with and without the lipase enzyme. The obtained samples were immediately frozen with liquid N_2_ and stored at −24 °C to stop the digestion process as each gastro-intestinal phase was finished (oral made up to 10 mL, gastric made up to 20 mL, and intestinal made up to 40 mL with MilliQ water for the analysis of bioactive compounds).

Betalains and phenolic compounds from each phase of in vitro gastro-intestinal digestion were analyzed following the method reported by Gómez-Maqueo et al. (2020) [23]. Aqueous samples were filtered with 0.45 µm syringe filters (E0032, Análisis Vínicos, Ciudad Real, Spain) into vials and analyzed by HPLC. Bioaccessibility was calculated as the ratio between the concentration of each bioactive compound in the intestinal fraction and the initial concentration in the *OPD* extract as reported Gómez-López et al. (2021a) [5].

Bioaccessibility (%) = (concentration in the intestinal fraction)/(initial concentration in the fruit) × 100.(1)

Additionally, stability (mg of bioactive compound/g of extract dry weight) and recovery (%, as a percentage of the concentration of the individual bioactive compound) in each digestion phase were compared to their initial contraction in the green OPD extract. 

### 2.7. Characteristics of Double Emulsions (W_1_/O/W_2_) 

#### 2.7.1. Encapsulation Efficiency 

The encapsulation efficiency (EE%) was measured as the quantity of each individual betalains and phenolic compounds that remained in the first emulsion (W_1_/O) after the double emulsification step at day 0. To carry out this analysis, 15 g of double emulsions (W_1_/O/W_2_) were centrifuged (Centrifuge 5804R, Eppendorf, Hamburg, Germany) at 12,880× *g* for 15 min at room temperature (24 °C) for Formulation A and at 2608× *g* during 10 min at room temperature (24 °C) for Formulation B. After centrifugation, 3× *g* of the inner emulsion (the supernatant) was recollected. Then, 5 mL of the chloroform:methanol:water mixture 60:10:30 (%, *v*/*v*/*v*) was added for Formulation A and the same components were added in the 67:23:10 proportion (%, *v*/*v*/*v*) for Formulation B. After that, the mixture was centrifuged for 3 min under the same conditions and the aqueous phase was removed. The solvents were removed with vacuum and the samples were stored at −24 °C until the HPLC analysis of bioactive compounds was carried out.

EE% was calculated by the following equation, where QBE is the quantity of individual bioactive compounds (betalains and phenolic compounds) achieved in 3 g of the inner emulsion after double emulsification step, and QB is the quantity of bioactive compounds that could be emulsified in 3 g of the inner emulsion (theoretical) from the used extract (control).

EE (%) = QBE/QB × 100.(2)

#### 2.7.2. Droplet Size and Distribution

Droplet size and distribution of W_1_/O/W_2_ formulations were measured by static light scattering technique using Mastersizer 3000 (Malvern Instruments Ltd., Malvern, UK) coupled to a Hydro system. Distilled water was used as the continuous phase, fixing a refractive index of 1.47. Data were reported as volume-weighted average (d4,3) and expressed in μm.

#### 2.7.3. Zeta Potential

Zeta potential was determined using Zetasizer Pro equipment (Malvern Instruments Ltd., Worcestershire, UK). A total of 100 μL of each formulation were diluted in 10 mL of MilliQ water and homogenized at 400 rpm. They were left settling for 5 min, and afterward they were transferred into disposable folded capillary cells to start the measurements. The samples were equilibrated for 120 s at 25 °C, and the analysis was conducted with a refraction index of 1.47, a scattering angle of 174° and distilled water as continuous phase. Measurements were carried out in triplicate on the same day as the preparation of the formulations. Data were expressed as mV.

#### 2.7.4. Physical Stability by Optical Inspection

The stability of double emulsions with encapsulated OPD extracts was measured according to Bou et al. (2014) [24] with modifications. It was determined in terms of gravitational phase separation (creaming) and recorded by triplicate for 28 days of storage at 7 °C. Stability was expressed as millimetres (mm) of the creaming height.

#### 2.7.5. Optical Microscopy

Optical microscopy was used to confirm that double emulsions were achieved in both formulations (A and B). Several drops of double emulsions were placed on a crystal slice and observed in the microscope. No dye was used to stain the samples. Optical microscopy was performed with a vertical microscope Axioskop (Carl Zeiss, Oberkochen, Germany) with Zeiss Plan-Neofluar lens (Carl Zeiss, Göttingen, Germany) coupled to a Leica DMC 6200 pixel-shift camera (Leica Microsystems, Wetzlar, Germany). The samples were observed with an open condenser and level four of illumination without colour filters. The samples were studied at 40× and at 100× with the addition of a drop of immersion oil to observe at 100×. 

#### 2.7.6. Confocal Laser Scanning Microscopy

Confocal laser scanning microscopy (CLSM) was used to observe the microstructure of the formulations with encapsulated OPD extracts before (control) and during the in vitro gastro-intestinal digestion process with lipase enzyme (oral, gastric, and intestinal phases). To stain the droplet structure (oil globules), 1 mL of control and each digestion phase were placed on a crystal slice. The samples were dyed by the addition of 10 µL Nile red solution (1 mg/mL in acetone). The excitation and emission for the fluorescence were measured at 488 and 523–650 nm, respectively. The samples were studied with a confocal multispectral TCS SP5 system (Leica Microsystems, Germany) at 20× and at 40× using a Zeiss Plan-Neofluar lens. 

#### 2.7.7. Colour Measurement

The colour of the double emulsions was measured using a UV–Vis Spectrophotometer, Specord 210 plus (Analytik-jena, Jena, Germany) with standard illuminant D65, 10° angle of illumination and spectral scan range from 380 to 780 nm. The CIELAB system was used to determinate the colour, where L* values represented lightness, a* values represented the green–red tonality and b* values represented the blue–yellow tonality. The samples were placed into disposable cuvettes and the colour was measured twice for each one.

### 2.8. Statistical Analysis

Data were expressed as mean ± standard deviation of at least two independent determinations (n = 2). Significant differences at a *p* < 0.05 significance level were calculated by an analysis of variance (ANOVA) with the SPSS Statistics software 26.0 for Windows (IBM corp., Armonk, New York, NY, USA). In addition, a Tukey test was used to make all of the pairwise comparisons between groups or samples.

## 3. Results and Discussion

### 3.1. Composition of Opuntia stricta var. dillenii (OPD) Green Extract

Betalains and phenolic compound profile of the OPD whole fruit green extracts and the encapsulated ones by Formulations A and B were analysed by HPLC according to Gómez-López et al. (2021a) [5]. The characterization of each compound was based on their retention time, UV–Vis and mass spectrum data. Table 2 shows the identification and quantification data of the most abundant bioactive compounds selected as target metabolites to study in this work, and Figure S1 (Supplementary Materials) shows the HPLC-DAD chromatograms of these extracts detected at 280 nm (for phenolic acids), 370 nm (for flavonoids), 480 nm (for betaxanthins) and 535 nm (for betacyanins). Table S1 (Supplementary Materials) shows all UV–vis and mass spectroscopy characteristics of the main individual betalains and phenolic compounds found in OPD green extracts which were studied.

The most abundant betalains in *Opuntia stricta* var. *dillenii*’s prickly pears were betacyanins, specially betanin (Peak 2) with a content of 5.41 ± 0.9 mg/g of dry weight, isobetanin (Peak 3) with a 3.71 ± 0.02 mg/g dry weight and neobetanin (Peak 4) with a 0.66 ± 0.01 mg/g dry weight (Table 2). In respect to phenolic acids, piscidic acid (Peak 1) was prevalent with a content of 7.29 ± 0.05 mg/g of dry weight. Regarding flavonoids, the most abundant were isorhamnetin glucoxyl-rhamnosyl-pentoside (IG2) (Peak 9) with a 0.47 ± 0.0 mg/g dry weight, and quercetin glycoside (QG2)-quercetin hexose pentoside (Peak 7) with a 0.17 ± 0.00 mg/g dry weight. Other flavonoids such as quercetin-3-O-rhamnosyl-rutinoside (QG3) (Peak 5), quercetin hexosyl pentosyl rhamnoside (QG1) (Peak 6) and isorhamnetin glucoxyl-rhamnosyl-rhamnoside (IG1) (Peak 8) were found at lower quantities of 0.13 ± 0.01 mg/g of dry weight, 0.13 ± 0.00 mg/g of dry weight and 0.05 ± 0.00 mg/g of dry weight, respectively (Table 2 and Table S1 (Supplementary Materials)).

### 3.2. Characterization of Double Emulsions (W_1_/O/W_2_)

#### 3.2.1. Droplet Morphology 

Both formulations (A and B) with encapsulated OPD green extracts showed a pink colour and a milky appearance, with L* = 26.73 ± 0.02, a* = 4.42 ± 0.24 and b* = 2.29 ± 0.37 CIELAB colour values for Formulation A; and L* = 32.40 ± 1.57, a* = 5.38 ± 1.02 and b* = 0.57 ± 0.30 for Formulation B. The appearance and colours were similar to those reported by Kaimainen et al. (2015) [20] in their study about Opuntia ficus-indica betalain encapsulation. Although both formulations have a similar concentration of the OPD extract, 0.31 ± 0.01 mg/mL W1 for Formulation A and 0.33 ± 0.01 mg/mL for Formulation B, Formulation B showed a pinker colour. According to the data, Formulation B showed a higher a* (red to green) value.

**Figure 2:**
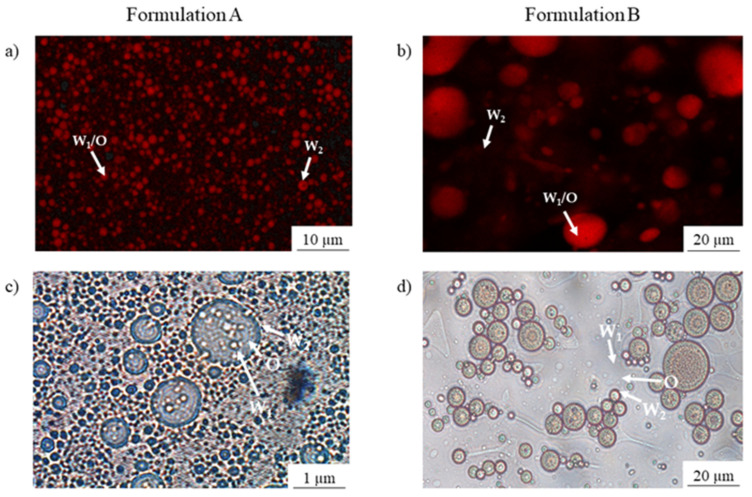
Optical and confocal microscopy images of double emulsion systems (W_1_/O/W_2_): (**a**) Confocal microscopy image of Formulation A; (**b**) Confocal microscopy image of Formulation B; (**c**) Optical microscopy image of Formulation A; (**d**) Optical microscopy image of Formulation B.

To confirm the formation of double emulsion, visual inspections with confocal microscopy and optical microscopy were performed. Figure 2a,b show the examination of the oil droplets into the continuous phase (Nile red staining) by confocal microscopy and Figure 2c,d show the images of the two phases of the inner emulsion by optical microscopy. The presence of oil droplets dispersed into the continuous phase (W_2_) (Figure 2a,b), and the Browian motion that could be observed by confocal microscopy verified the formation of the double emulsion between control and formulations.

The smaller drops of the OPD extract (W_1_) that could be observed inside the oil phase (O) (Figure 2c,d) also proved the formation of the double emulsions (W_1_/O/W_2_) [25]. Ding et al. (2019) [26] in their review about double emulsion morphologies reported that they can be classified according to the number of water droplets (W_1_) contained in the inner emulsion (W_1_/O): microcapsules (only one water droplet includes the oil droplet), multivesicular (more than one water droplet into oil droplet) and microspheres (like the previous one but with a complex inner structure inside oil droplet). Our results suggested the formation of microsphere-type double emulsions in both formulations (Figure 2). Similar observations were reported in the double emulsion encapsulation of bioactive compounds from cactus acid fruit by a two-stage method [27]. 

#### 3.2.2. Droplet Size Distribution and Zeta Potential

**Figure 3:**
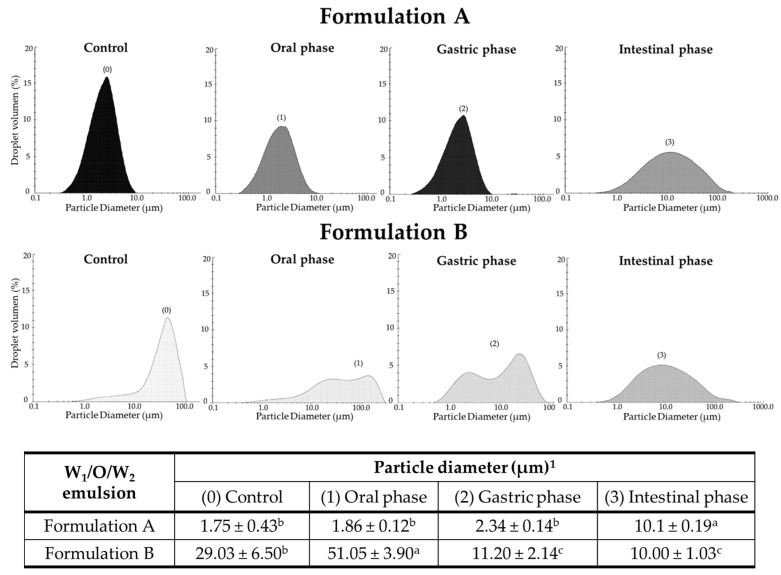
Droplet size and particle size distribution of Formulation A and Formulation B with encapsulated OPD green extract (0) (control, before digestion) and during the in vitro gastro-intestinal digestion: (1) oral phase; (2) gastric phase; (3) intestinal phase.

Emulsifiers play an important role in the formation of double emulsions as they facilitate the production of the emulsion droplets during homogenization and can help the reduction of the interfacial tension [28]. Moreover, depending on the nature of the surfactant, the size of the drops might change, small sizes being the most appropriate as they result in a more stable system. Figure 3 shows that Formulation A, made with Tween 20 (nonionic surfactant) as the secondary phase surfactant (Table 1), had the lowest droplet size (1.75 ± 0.43 µm). The same behaviour was reported by Velderrain-Rodriguez et al. (2019) [19] in their research about phenolic compounds encapsulation, obtaining the lowest particle size also using Tween 20 as a surfactant. Nonionic surfactants are commonly used to produce small droplet emulsions (nanoemulsions or microemulsions) [28]. On the other hand, Walstra (2002) [29] reported that using proteins as surfactants was not as good for forming fine emulsions as they are less effective at reducing interfacial tension, and so they form larger droplets [30]. These facts were concurrent with the obtained data of the present study shown in Figure 3. Here, Formulation B, which was made with sodium caseinate (protein surfactant), showed a higher droplet size (29.03 ± 6.5 µm). Droplet size distribution can also be seen in Figure 3, showing that the size of the oil droplets in Formulation B remains higher than in Formulation A in the confocal microscopy images.

Zeta potential is the measurement of the physical interactions of repulsion or attraction between charged particles in a suspension, and it allows the prediction of surface interactions (dispersion, aggregation, or flocculation) and long-term stability [31]. Regarding emulsions, it highly depends on the chemical nature of the polymer, the stabilisers, and the pH of the matrix [32]. In case of Formulation A, the average zeta potential was −32.7 ± 1.1 mV, while in Formulation B it was −42.9 ± 3.8 mV. According to Honary and Zahir (2013) [33], the absolute zeta potential values over 30 mV suggest that the emulsion system is stable, as high interactions between particles are occurring, to which both Formulation A and B conform. 

Similar values (between −26 mV and −47 mV) were obtained by Ribeiro et al. (2015) [34], who encapsulated hydroglycolic extracts from *Opuntia ficus-indica* by simple emulsion systems using anionic surfactants, too. ^1^ Results were expressed as mean ± standard deviation. This came from obtaining at least two independent measurements (n = 2) of each sample. Superscript lower-case letters indicate statistically significant differences (*p* ≤ 0.05) between the digestion phases of each formulation.

#### 3.2.3. Encapsulation Efficiency 

Encapsulation efficiency values (EE%) ranged from 98% to 68.2%, depending on the formulation and bioactive compound, Table 3. Regarding betalains, the most efficiently encapsulated compound was betanin by Formulation A (96.9 ± 3.3%) and isobetanin by Formulation B (98 ± 3.9%). Piscidic acid showed values of 71 ± 1.3% in Formulation A and 70.2 ± 5.7% in Formulation B, with no significant statistical differences between them. Isorhamnetin glucoxyl–rhamnosyl–rhamnoside (IG1) was the flavonoid with the higher EE% in Formulation A (89.9 ± 1.4%) and Isorhamnetin glucoxyl–rhamnosyl–pentoside (IG2) in Formulation B (95.9 ± 7.7%). Overall, the encapsulation was more efficient for Formulation B, which could be due to the use of gelatine in its processing since, as reported by Robert et al. (2020) [16], the gelling of the internal or the external aqueous phase of a double emulsion increases the stability of the system as the particle movement is reduced. In addition, as mentioned in the research by Jang and Koh (2023) [35], the use of anionic polysaccharides, such as Arabic gum and xanthan gum, lead to an increased stability of encapsulated formulations. In general terms, betalains showed the highest encapsulation efficiency (EE%) of 93.2 ± 2.2 and 96.6 ± 1.8 for Formulations A and B, respectively, followed by flavonoids with 85.8 ± 2,7 and 91.5 ± 7.9 for Formulations A and B, respectively, and finally piscidic acid with 71 ± 0.9 and 70.2 ± 4.6 for Formulations A and B, respectively. These differences in EE (%) between the different families of bioactive compounds could be attributed to the distinct interactions between betalains and phenolic compounds within OPD extracts and the ingredients used to formulate each type of double emulsion (W_1_/O/W_2_). Kaimanen et al. (2014) [20] reported the encapsulation efficiency of double emulsions encapsulating betalain compounds from red beet extracts, showing EE (%) values between 87.2 and 90.1%. Other authors described the encapsulation of total phenolics from *Opuntia xoconostle* extracts by single and double emulsions, obtaining values of encapsulation efficiency between 66.32 and 95.91% [27].

#### 3.2.4. Physical Stability

**Figure 4:**
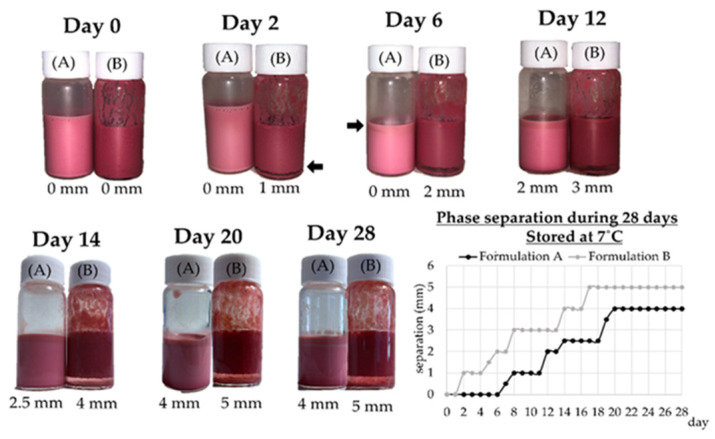
Colour and stability of *Opuntia stricta* var. *dillenii* double emulsions (W_1_/O/W_2_) (Formulations A and B) during storage at 7 °C.

Instability phenomena lead to changes in the steadiness of double emulsions that could be seen by optical inspection such as gravitational separation or creaming analysis. Figure 4 shows the gravitational separation of both double emulsions (W_1_/O/W_2_), Formulations A and B, during 28 days of storage at 7 °C. Formulation A remained stable until Day 6, when creaming began. This creaming increased until Day 20, and then kept steady with 4 mm of separation between the two observed phases. Formulation B maintained the initial structure only within the first 2 days, and after this, the creaming enlarged. By Day 17 it achieved stability, with a phase separation of 5 mm. Regarding droplet size, Formulation B had larger droplets than formulation A, so this could be the reason for the sooner start of its creaming, although the zeta potential values suggested that Formulation B could be more stable. Walstra (2002) [29] reported that larger droplets cause faster creaming in the emulsion systems, and this observation coincides with the obtained results in this study. The incorporation of compounds that modify the formulation viscosities, such as Arabic and xanthan gums, delays the collision between droplets and could increase the stability of the double emulsion [36]. In our case, although Formulation B contained guar and Arabic gum, it turned unstable more rapidly.

### 3.3. In Vitro Gastro-Intestinal Digestion of Double Emulsions (W_1_/O/W_2_)

#### 3.3.1. Microstructure of W_1_/O/W_2_ Formulations during In Vitro Gastro-Intestinal Digestion

**Figure 5:**
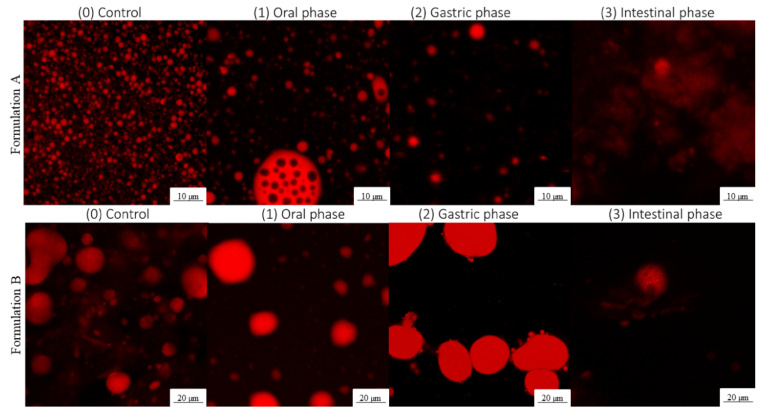
Confocal microscopy images of the double emulsion systems during in vitro gastro-intes-tinal digestion: the integrity of the formulations can be seen on the control (0) and oral phase (1), the aggregation of the W_1_/O/W_2_ droplets in the gastric phase (2) and the complete coalescence in the intestinal phase (3).

Confocal microscopy images of the formulations along the gastro-intestinal digestion with the use of lipase enzyme are shown in Figure 5.

In Formulation A, an increase in particle size was visible as the gastro-intestinal digestion progressed. This fact could be attributed to the action of enzymes and digestion fluids, causing coalescence, aggregation, and phase break-up phenomena. The initiation of double emulsion digestion lay in the “disassembly” of the W_2_ phase, which allowed lipolytic enzymes to gain access to the fat globules of the oil phase [37]. This was followed by a reduction in the lipid barrier through lipase digestion and solubilisation of the digestion products into bile salt micelles, leading to coalescence of the finer emulsion particles [20]. Therefore, in the gastric phase, an increase in particle diameter was observed with respect to the oral phase, and in the intestinal phase it was observed with respect to the gastric phase (Figure 5).

These data agree with those obtained by Kaimainen et al. (2014) [20], who analysed the particle size and distribution of betalain-rich double emulsions at different times of the digestion process and reported an enlargement throughout the process. The study of the intestinal phase showed that the emulsion particles were digested and degraded, undergoing a loss of the original structure that lead to the release of the bioactive compounds, such as betalains and phenolic compounds. 

With respect to Formulation B, the reverse process took place, i.e., a decrease in particle size through the gastro-intestinal digestion process was observed. It must be considered that in Formulation B, the starting value of particle size was considerably larger (20.03 ± 6.50 µm) than that found for Formulation A (1.75 ± 0.43 µm) (Figure 3), so the formation of the aggregates between the compounds might have taken place, as Formulation B had a more viscous and consistent nature. However, at the end of the digestion, the intestinal phase, it had very similar characteristics and particle size (10.00 ± 1.03 µm) to those of Formulation A.

#### 3.3.2. Digestive Stability and Bioaccessibility of Betalains and Phenolic Compounds Encapsulated by Double Emulsions (W_1_/O/W_2_)

Table 4 shows the bioaccessibility and stability values obtained for the main individual bioactives, betalains and phenolic compounds (Table 2), from OPD green extracts (control) and the encapsulated ones by the two double emulsion formulations after the in vitro gastro-intestinal digestion, with and without the use of the gastric lipase enzyme. The use of gastric lipase enzyme in the digestion assay was performed in order to elucidate its influence on the degradation of the oil phase of the formulations and, for this reason, on the bioaccessibility and stability of encapsulated bioactives. Table S3 (Supplementary Materials) shows the recovery percentage of the main bioactives along gastro-intestinal digestion. 

Bioaccessibility values (expressed in percentage) of the encapsulated OPD bioactives by Formulations A and B were higher in comparison to the values obtained for non-encapsulated extracts, indicating the efficient protection of these bioactive compounds by the double emulsions (W_1_/O/W_2_) after exposure to in vitro gastro-intestinal digestion, mostly for Formulation A. Betanin was the most bioaccessible betalain, mainly when the green *OPD* extract was encapsulated with Formulation A (93.4 ± 0.9% *w*/*o* lipase; 70.8 ± 1.8% *w*/ lipase). Other betacyanin, neobetanin, behaved as an exception, showing the non-encapsulated extract values (47.2 ± 0.1%) higher than those obtained for the formulations, even though it had been efficiently encapsulated (73.7 ± 6.7 EE% in Formulation A and 95.2 ± 3.2 EE% in Formulation B). In the results reported by Gómez-López et al. (2021a) [5], this behaviour was also observed; neobetanin had significantly lower bioaccessibility values than the other bioactive compounds, suggesting that this bioactive compound may be particularly sensitive to the gastro-intestinal conditions. Piscidic acid showed higher bioaccessibility values on the encapsulated OPD extracts, especially for Formulation A (253.1 ± 5.8% *w*/*o* lipase; 251.9 ± 4.7% *w*/ lipase). Similarly, Santiago et al. (2018) [38] and Missaoui et al. (2020) [39] reported similar behaviours on the very high bioaccessibility of piscidic acid in *O. ficus-indica* extracts, over 150% and 205%, respectively, even though they were not encapsulated extracts. This fact could be explained by the partial association that occurs between the polyphenols and the dietary fibres of the fruit as they present hydroxyl groups (−OH) that give rise to hydrogen bonds and Van der Waals interactions, i.e., non-covalent bonds, which, when subjected to the digestion conditions, hydrolyse and allow the release of the compounds, causing a positive increase in their bioaccessibility. Furthermore, some isomerization reactions might be occurring among the stereoisomeric forms of the different isomers of piscidic acids present in the *OPD* extracts, which can lead to the observed quantitative changes [39]. With respect to flavonoids, although only a few were encapsulated with considerable EE% values, only Isorhamnetin glucoxyl–rhamnosyl–pentoside (IG2) was detected in the intestinal phase, particularly in Formulation A (91.1 ± 2.8% *w*/*o* lipase; 75.7 ± 3.8% *w*/ lipase), Table 4. This is a logical fact considering that it is the most abundant flavonoid in OPD whole fruit extracts (Table 2). The other flavonoid compounds present in the OPD extracts may not have been detected due to the dilution at which they are found in the intestinal phase, or because they have been degraded during the process. To confirm this, it would probably be necessary to carry out a study of the gastro-intestinal digestion with formulations with higher concentrations of OPD green extracts. 

It should be noted that bioaccessibility values remained higher when the assay was performed without using the lipase enzyme, but, as stated in the experimental design of the INFOGEST in vitro digestion protocol [22], it is highly recommended to take into account the influence of this enzyme in the process, especially if the samples have a considerable fat content, because gastric lipolysis not only contributes to the overall digestion of triacylglycerols, but also induces the subsequent action of pancreatic lipase on lipid substrates that may be poorly digested by pancreatic lipase alone. Applying this concept to the gastro-intestinal digestion of the formulations under study, it was necessary to use gastric lipase to make the digestion of the oil phase as realistic as possible in order to determine which fraction of encapsulated OPD bioactives was released at the end of the intestinal phase. In relation to the stability (data shown in Table 4), where values are expressed in mg of compound/g of dry extract, the content of all bioactive compounds in the non-encapsulated OPD extracts decreased throughout the gastro-intestinal digestion process, particularly at the intestinal phase, where they were reduced to less than half compared to the control, except for piscidic acid, which remained slightly above. Meanwhile, the stability values for the same compounds encapsulated in Formulations A and B (encapsulated extracts) also suffered a decrease throughout digestion as occurred in control samples, but this was less noticeable. In fact, some bioactive compounds showed higher content values in the different phases of digestion than in the control (non-encapsulated extract). This was especially notable for piscidic acid, a compound for which increases of more than double of its concentration in the intestinal phase were obtained. This fact was explained before, when piscidic acid bioaccessibility was discussed. As an exception, only neobetanin showed a more pronounced declining through the gastro-intestinal digestion on both formulations than on the unencapsulated extract (control). Moreover, it should be noted that neobetanin was not detected in the lipase gastric phase of Formulation A, but it was identified in the oral and intestinal phases, probably due to the low amounts in all phases that complicated its quantification. These results can also be seen expressed in recovery percentages in Table S3 (Supplementary Materials).

## 4. Conclusions

In this study, two formulations (A and B) based on double emulsions (W_1_/O/W_2_) were assayed to encapsulate green extracts from *Opuntia stricta* var. *dillenii* whole fruits, abundant in betalains and phenolic compounds. Formulation A, based on Tween 20, showed lower droplet sizes (1.75 µm) and remained more stable during storage in comparison with Formulation B (29.03 µm) based on caseinate (protein emulsifier). Although the zeta potential for both formulations indicated that they were both stable systems (values above 30 in absolute value), only Fomulation B was predicted to be more stable. Regarding the encapsulation efficiency, betalains were the OPD bioactives that showed higher values (from 98 to 73.7%) depending on the compound and formulation, followed by flavonoids (68.2 to 96.6%) and piscidic acid (70.2 to 71%). In general, Formulation B, based on the use of casein, showed greater encapsulation efficiencies for OPD bioactives. For both Formulations, A and B, the digestive stability and bioaccessibility of the OPD green extract bioactives (with and without lipase enzyme) were significantly higher than in the non-encapsulated green extracts (values from 61.7 to 253.1%), with the exception of neobetanin that showed lower values. These results suggested that both assayed double emulsions could be suitable as efficient microcarrier systems for betalains and phenolic compounds from OPD extracts along the digestive process, as they maintained the emulsion structure until the intestinal phase, in a great efficacy formulation A (based in the use of Tween 20). 

## Figures and Tables

**Table 1:** Composition of double emulsions W_1_/O/W_2_ to encapsulate *Opuntia stricta* var. *dillenii* green extracts.

Type of Emulsion			Reagent and Solutions ^1^
Formulation A W_1_/O/W_2_	Primary emulsion (W_1_/O) 25%	Aqueous phase (W_1_) 25%	NaCl (0.1 M)
			Glycerol
			*OPD* extract
		Oil phase (O) 75%	MCT oil
			PGPR
	Aqueous phase (W_2_) 75%		NaCl (0.1 M)
			Tween 20 (2%)
Formulation B W_1_/O/W_2_	Primary emulsion (W_1_/O) 30%	Aqueous phase (W_1_) 30%	Gelatine 6% in TRIS buffer (pH 9)
			*OPD* extract
		Oil phase (O) 70%	MCT oil
			PC
			PGPR
	Aqueous phase (W_2_) 70%		Caseinate at 3%
			NaCl 10% in glycerol
			Guar gum
			Acacia gum (Arabic)

^1^ NaCl: sodium chloride; *OPD*: *Opuntia stricta* var. *dillenii* green extract; MCT oil: Caprylic acid triglyceride oil; PGPR: polyglycerol polyricinoleate; TRIS buffer: Tris(hydroxymethyl) aminomethane; Tween 20: Polysorbate 20; PC: Phosphatidylcholine.

**Table 2:** Content in the main individual bioactive compounds present in *Opuntia stricta* var. *dillenii.* green extracts (Control) and encapsulated by formulations (A and B).

Peak ^1^	Compound	Content (mg/g Dry Weight) ^2^		
		Control ^3^	Formulation A ^4^	Formulation B ^4^
1	Piscidic acid	7.29 ± 0.05	2.00 ± 0.04 ^a^	1.98 ± 0.16 ^a^
2	Betanin	5.41 ± 0.9	2.91 ± 0.22 ^a^	2.83 ± 0.08 ^a^
3	Isobetanin	3.71 ± 0.02	1.79 ± 0.02 ^a^	1.95 ± 0.15 ^a^
4	Neobetanin	0.66 ± 0.01	0.36 ± 0.06 ^b^	0.47 ± 0.06 ^a^
5	Quercetin-3-O-rhamnosyl-rutinoside (QG3)	0.13 ± 0.01	0.09 ± 0.02 ^a^	0.04 ± 0.02 ^b^
6	Quercetin hexosyl pentosyl rhamnoside (QG1)	0.13 ± 0.0	0.05 ± 0.02 ^a^	0.03 ± 0.0 ^a^
7	Quercetin hexose pentoside (QG2)	0.17 ± 0.0	0.13 ± 0.02 ^a^	0.05 ± 0.02 ^b^
8	Isorhamnetin glucoxyl-rhamnosyl-rhamnoside (IG1)	0.05 ± 0.0	0.01 ± 0.0 ^a^	0.01 ± 0.0 ^a^
9	Isorhamnetin glucoxyl-rhamnosyl-pentoside (IG2)	0.47 ± 0.05	0.19 ± 0.02 ^b^	0.31 ± 0.04 ^a^

^1^ Peak numbers are presented according to Table S1, Supplementary Materials. ^2^ Results are expressed as mean ± standard deviation. Superscript letters indicate statistically significant differences (*p* ≤ 0.05). ^3^ Control refers to the green extracts obtained from *Opuntia dillenii*’s whole fruits. ^4^ Formulation A based on Tween 20 and Formulation B based on caseinate.

**Table 3:** Encapsulation efficiency of individual main betalains and phenolic compounds from encapsulated OPD green extracts by double emulsions (W_1_/O/W_2_), Formulations A and B.

	Encapsulation Efficiency (%) ^1^	
Compound	Formulation A ^2^	Formulation B ^2^
BETALAINS		
Betanin	96.9 ± 3.3 ^a^	94.2 ± 2.5 ^a^
Isobetanin	92.2 ± 0.1 ^a^	98 ± 3.9 ^a^
Neobetanin	73.7 ± 6.7 ^b^	95.2 ± 3.2 ^a^
PHENOLIC ACIDS		
Piscidic acid	71 ± 1.3 ^a^	70.2 ± 5.7 ^a^
FLAVONOIDS		
Quercetin-3-O-rhamnosyl-rutinoside (QG3)	87.7 ± 3.1 ^a^	88.5 ± 1.5 ^a^
Quercetin hexosyl pentosyl rhamnoside (QG1)	89.7 ± 3.2 ^a^	81.4 ± 1.5 ^b^
Isorhamnetin glucoxyl–rhamnosyl–rhamnoside (QG2)	84.4 ± 6.2 ^a^	68.2 ± 5.9 ^b^
Isorhamnetin glucoxyl–rhamnosyl–rhamnoside (IG1)	89.9 ± 1.4 ^a^	91.7 ± 3.4 ^a^
Isorhamnetin glucoxyl–rhamnosyl–pentoside (IG2)	74.2 ± 3.6 ^b^	95.9 ± 7.7 ^a^
Total Betalains	93.2 ± 2.2 ^a^	96.6 ± 1.8 ^a^
Total Phenolic Acids	71 ± 1.3 ^a^	70.2 ± 5.7 ^a^
Total Flavonoids	85.8 ± 2.7 ^a^	91.5 ± 7.9 ^a^

^1^ Results re expressed as mean ± standard deviation. Superscript letters indicate statistically significant differences (*p* ≤ 0.05) between formulations. ^2^ Formulation A based on Tween 20 and Formulation B based on caseinate.

**Table 4:** Digestive stability and bioaccessibility of the main betalains and phenolic compounds from *Opuntia stricta* var. *dillenii* green extracts and Formulation A and Formulation B after in vitro gastro-intestinal digestion.

	Stability (mg Comp./g of Dry Extract) ^1^							Bioaccessibility (%) ^1^			
Compounds	In Vitro Phase	Extract ^1^	Formulation A ^2^		Formulation B ^3^		Control ^1^	Formulation A ^2^		Formulation B ^3^	
			*w*/*o* Lipase	*w*/ Lipase	*w*/*o* Lipase	*w*/ Lipase		*w*/*o* Lipase	*w*/ Lipase	*w*/*o* Lipase	*w*/ Lipase
BETALAINS											
Betanin	Control	5.41 ± 0.09	1.39 ± 0.23 ^Ab^	1.41 ± 0.3 ^Ab^	2.88 ± 0.08 ^Aa^	2.79 ± 0.2 ^Aa^	-	-	-	-	-
	Oral	4.91 ± 0.02	1.73 ± 0.28 ^Ab^	1.46 ± 0.05 ^Ab^	3.20 ± 0.14 ^Aa^	2.55 ± 0.22 ^Ba^	-	-	-	-	-
	Gastric	4.67 ± 0.12	1.54 ± 0.06 ^Ab^	1.15 ± 0.08 ^Bb^	2.01 ± 0.04 ^Aa^	2.10 ± 0.58 ^Aa^	-	-	-	-	-
	Intestinal	1.89 ± 0.01	1.31 ± 0.19 ^Ab^	0.99 ± 0.2 ^Bb^	1.89 ± 0.05 ^Aa^	1.59 ± 0.05 ^Aa^	35.3 ± 0.2	93.4 ± 0.9 ^Aa^	70.8 ± 1.8 ^Ba^	67.1 ± 0.1 ^Ab^	56.3 ± 0.2 ^Bb^
Isobetanin	Control	3.71 ± 0.02	1.1 ± 0.17 ^Ab^	1.08 ± 0.1 ^Ab^	1.96 ± 0.08 ^Aa^	1.95 ± 0.1 ^Aa^	-	-	-	-	-
	Oral	2.97 ± 0.01	1.39 ± 0.05 ^Ab^	0.96 ± 0.18 ^Bb^	2.03 ± 0.03 ^Aa^	1.72 ± 0.22 ^Ba^	-	-	-	-	-
	Gastric	2.56 ± 0.0	1.04 ± 0.06 ^Ab^	0.79 ± 0.0 ^Bb^	1.34 ± 0.08 ^Aa^	1.31 ± 0.27 ^Aa^	-	-	-	-	-
	Intestinal	1.12 ± 0.0	0.88 ± 0.15 ^Ab^	0.66 ± 0.09 ^Bb^	1.32 ± 0.07 ^Aa^	1.08 ± 0.01 ^Ba^	30.1 ± 0.1	90.9 ± 0.9 ^Aa^	68.1 ± 0.6 ^Ba^	67.7 ± 1 ^Ab^	55.8 ± 3.6 ^Bb^
Neobetanin	Control	0.66 ± 0.01	0.25 ± 0.01 ^Ab^	0.23 ± 0.01 ^Ab^	0.5 ± 0.1 ^Aa^	0.47 ± 0.06 ^Aa^	-	-	-	-	-
	Oral	0.35 ± 0.0	0.17 ± 0.02 ^Ab^	0.19 ± 0.01 ^Aa^	0.28 ± 0.01 ^Aa^	0.18 ± 0.02 ^Ba^	-	-	-	-	-
	Gastric	0.32 ± 0.03	0.14 ± 0.04 ^Ab^	n.d.	0.25 ± 0.01 ^Aa^	0.09 ± 0.02 ^B^	-	-	-	-	-
	Intestinal	0.31 ± 0.0	0.11 ± 0.03 ^Ab^	0.08 ± 0.0 ^Aa^	0.21 ± 0.02 ^Aa^	0.05 ± 0.01 ^Bb^	47.2 ± 0.1	59.9 ± 9 ^Aa^	41.7 ± 1 ^Ba^	44.5 ± 1.9 ^Ab^	11.5 ± 0.7 ^Bb^
PHENOLIC ACIDS											
Piscidic acid	Control	7.29 ± 0.05	4.43 ± 0.1 ^Aa^	4.39 ± 0.02 ^Aa^	2.27 ± 0.05 ^Ab^	2.23 ± 0.09 ^Ab^	-	-	-	-	-
	Oral	7.01 ± 0.27	2.08 ± 0.05 ^Ab^	2.06 ± 0.29 ^Aa^	2.64 ± 0.13 ^Aa^	2.01 ± 0.57 ^Ba^	-	-	-	-	-
	Gastric	7.01 ± 0.24	3.44 ± 0.03 ^Aa^	1.84 ± 0.4 ^Bb^	2.52 ± 0.02 ^Ab^	2.06 ± 0.85 ^Ba^	-	-	-	-	-
	Intestinal	4.69 ± 0.21	10.99 ± 0.5 ^Aa^	10.94 ± 0.1 ^Aa^	5.06 ± 0.17 ^Bb^	8.48 ± 2.86 ^Ab^	64.3 ± 3.3	253.1 ± 5.8 ^Aa^	251.9 ± 4.7 ^Aa^	226.9 ± 1 ^Bb^	247.3 ± 5.4 ^Aa^
FLAVONOIDS											
Isorhamnetin glucoxyl-rhamnosyl-pentoside (IG2)	Control	0.47 ± 0.0	0.2 ± 0.01 ^Ab^	0.19 ± 0.01 ^Ab^	0.3 ± 0.05 ^Aa^	0.31 ± 0.03 ^Aa^	-	-	-	-	-
	Oral	0.35 ± 0.01	0.21 ± 0.02 ^Ab^	0.17 ± 0.01 ^Ba^	0.28 ± 0.02 ^Aa^	0.19 ± 0.0 ^Ba^	-	-	-	-	-
	Gastric	0.33 ± 0.0	0.18 ± 0.01 ^Ab^	0.17 ± 0.0 ^Aa^	0.22 ± 0.01 ^Aa^	0.19 ± 0.03 ^Ba^	-	-	-	-	-
	Intestinal	0.11 ± 0.0	0.18 ± 0.01 ^Aa^	0.15 ± 0.01 ^Ba^	0.19 ± 0.0 ^Aa^	0.14 ± 0.01 ^Ba^	23.5 ± 0.3	91.1 ± 2.8 ^Aa^	75.7 ± 3.8 ^Ba^	58.7 ± 0.5 ^Ab^	46.2 ± 5.3 ^Bb^

^1^ Results are expressed as mean ± standard deviation. Superscript capital letters indicate statistically significant differences (*p* ≤ 0.05) between gastro-intestinal digestion phases without or with lipase within the same formulation. Superscript lower-case letters indicate statistically significant differences (*p* ≤ 0.05) between the different formulations under the same conditions of using or not using lipase. ^2^ Extract refers to the green extracts obtained from *Opuntia stricta* var. *dillenii*’s whole fruits. ^3^ Formulation A based on Tween 20 and Formulation B based on caseinate.

## Data Availability

Data is contained within the article (or supplementary material).

## Supplementary Materials

The following supporting information can be downloaded at: https://www.mdpi.com/article/10.3390/foods12112243/s1, Figure S1: HPLC chromatograms of betalains and phenolic compounds from *Opuntia stricta* var. *dillenii* green extracts. The numbers associated to the peaks are according to Table S1; Table S1: Characterization of the main betalains and phenolic compounds present in *Opuntia stricta* var. *dillenii* green extracts analyzed by HPLC-DAD and HPLC-MS1 [5]; Table S2: Composition of the digestive phases and the enzymes used for the in vitro gastro-intestinal digestion assay by INFOGEST method; Table S3. Recovery of main betalains and phenolic compounds from *Opuntia stricta* var. *dillenii* green extracts (control) encapsulated by Formulation A and Formulation B after in vitro gastro-intestinal digestion.
