# Supplementary material for: Oil-Based Double Emulsion Microcarriers for Enhanced Stability and Bioaccessibility of Betalains and Phenolic Compounds from Opuntia stricta var. dillenii Green Extracts
Source: Foods. 2023 Jun 1;12(11):2243. doi: 10.3390/foods12112243 (PMC10253192; doi:10.3390/foods12112243)
Supplement: Supplementary file 1 [file foods-12-02243-s001.zip › foods-2398133-supplementary final.pdf]

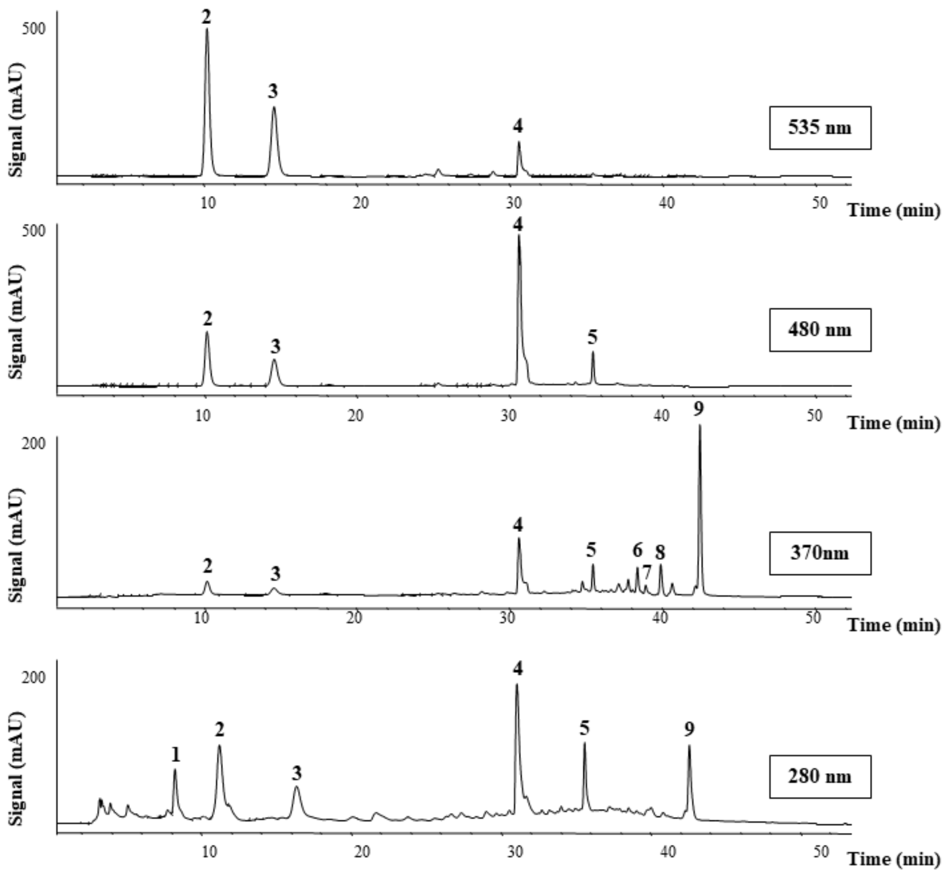

Figure S1. HPLC chromatograms of betalains and phenolic compounds from *Opuntia stricta* var. *dillenii* green extracts. The numbers associated to the peaks are according to Table S1.

Table S1. Characterization of the main betalains and phenolic compounds present in *Opuntia stricta* var. *dillenii* green extracts analyzed by HPLC-DAD and HPLC-MS[1].

Peak	t_R (min)	Compound	UV λ max (nm)	$[M\text{-}H]^+$	$[M\text{-}H]^-$	MS/MS (m/z)
**1**	8.83	Piscidic acid	272		255	193, 165, 135, 119, 107
**2**	10.76	Betanin	535	551		390, 389
**3**	15.23	Isobetanin	535	551		390, 389
**4**	31.34	Neobetanin	467		549	387
**5**	35.14	Quercetin-3-O-rhamnosyl-rutinoside (QG3)	358	757		611, 303
**6**	38.65	Quercetin hexosyl pentosyl rhamnoside (QG1)	255, 358	426		303, 191, 120
**7**	39.56	Quercetin glycoside (QG2)-Quercetin hexose pentoside	255, 353	653		303, 177
**8**	40.00	Isorhamnetin glucoxyl-rhamnosyl-rhamnoside (IG1)	256, 356	771		625, 317, 85
**9**	42.57	Isorhamnetin glucoxyl-rhamnosyl-pentoside (IG2)	254, 356	757		317, 167, 86

[1]Data were previously published by Gómez-López et al, 2021 [5].

Table S2. Composition of the digestive phases and the enzymes used for the *in vitro* gastrointestinal digestion assay by INFOGEST method.

Reagents	Stock	Simulated oral phase	Simulated gastric phase	Simulated intestinal phase
Simulated digestive phases at 1:1 (w/w)				
		% (v/v)		
KCl	0.5 M	2	5.6	2.16
KH_2PO_4	0.5 M	4	0.18	0.32
$NaHCO_3$	1 M	0.8	2.6	17.0
NaCl	2 M	0.2	2	3.2
$MgCl_2(H_2O)_6$	0.15 M	0.2	0.4	0.44
HCl	1 M	0.8	0.6	0.2
NaOH	1 M	0.2	-	0.32
MilliQ water	-	91.8	88.62	76.36
pH		6.8	1.3	8.2
Addition of enzyme % (v/v)				
Salivary amylase	75 U/ml	5	-	-
Pepsine	2500 U/ml	-	4 - 8*	-
Lipase	25 U/ml	-	4	-
Pancreatine	100 U/ml	-	-	12
Bile salt	10 Mm	-	-	6

* Only added when the assay was performed with lipase.

Table S3. Recovery of main betalains and phenolic compounds from *Opuntia stricta* var. *dillenii* green extracts (control) encapsulated by Formulation A and Formulation B after *in vitro* gastro-intestinal digestion.

		Recovery (%)[1]				
**Compound**	**In vitro phase**	**OPD green Extract[2]**	**Formulation A[3]**		**Formulation B[3]**	
			w/o lipase	w/ lipase	w/o lipase	w/ lipase
**BETALAINS**						
Betanin	Oral	91.7 ± 0.3	114.5 ± 3.4^{Aa}	106.5 ± 14.9^{Aa}	113.1 ± 1.3^{Aa}	90.1 ± 7.1^{Ba}
	Gastric	87.8 ± 2.2	108.4 ± 4.4^{Aa}	83.1 ± 5.9^{Ba}	71.1 ± 0.4^{Ab}	74.1 ± 13^{Aa}
	Intestinal	35.3 ± 0.2	93.4 ± 0.9^{Aa}	70.8 ± 1.8^{Ba}	67.1 ± 0.1^{Ab}	56.3 ± 0.2^{Bb}
Isobetanin	Oral	79.8 ± 0.2	123.5 ± 5.5^{Aa}	101.6 ± 1.8^{Ba}	104.7 ± 4.7^{Ab}	88.2 ± 3.3^{Bb}
	Gastric	68.8 ± 0.1	99.9 ± 3.9^{Aa}	81.7 ± 8.5^{Aa}	69.3 ± 6.6^{Ab}	68.1 ± 13.5^{Aa}
	Intestinal	30.1 ± 0.1	90.9 ± 0.9^{Aa}	68.1 ± 0.6^{Ba}	67.7 ± 1^{Ab}	55.8 ± 3.6^{Bb}
Neobetanin	Oral	52.6 ± 0.6	84.6 ± 1.6^{Ba}	93.7 ± 4.9^{Aa}	60 ± 7.5^{Ab}	38.5 ± 6.9^{Bb}
	Gastric	48.1 ± 4.3	69.3 ± 5.2	n.d.	53.6 ± 6.2^{A}	18.8 ± 1.2^{B}
	Intestinal	47.2 ± 0.1	52.9 ± 9.1^{Aa}	41.7 ± 1^{Ba}	44.5 ± 1.9^{Ab}	11.5 ± 0.7^{Bb}
**PHENOLIC ACIDS**						
Piscidic acid	Oral	96.1 ± 4.4	47.9 ± 2.1^{Ab}	47.5 ± 5.5^{Ab}	118.3 ± 0.8^{Aa}	77.5 ± 1.1^{Ba}
	Gastric	96.1 ± 3.9	79.2 ± 1.2^{Ab}	42.5 ± 7.2^{Bb}	113.2 ± 2.5^{Aa}	128.2 ± 1.4^{Ba}
	Intestinal	64.3 ± 3.3	253.1 ± 5.8^{Aa}	251.9 ± 4.8^{Aa}	226.9 ± 0.9^{Ab}	247.3 ± 5.4^{Ba}
**FLAVONOIDS**						
Isorhamnetin glucoxyl-rhamnosyl-pentoside (IG2)	Oral	73.9 ± 2.4	107.7 ± 3.5^{Aa}	87.1 ± 0.1^{Ba}	81.9 ± 4.4^{Ab}	62.58 ± 4.9^{Bb}
	Gastric	68.7 ± 0.6	92.1 ± 6.7^{Aa}	84.2 ± 2.5^{Aa}	65.5 ± 2.8^{Ab}	62.4 ± 12.6^{Ab}
	Intestinal	23.5 ± 0.3	91.1 ± 2.8^{Aa}	75.7 ± 3.8^{Ba}	58.7 ± 0.5^{Ab}	46.2 ± 5.3^{Bb}

[1]Results were expressed as mean ± standard deviation. This came from obtaining at least two independent measurements (n= 2) of each sample. Superscript capital letters indicate statistically significant differences ($p \leq 0.05$) between gastro-intestinal digestion phases without or with lipase within the same formulation. Superscript lower-case letters indicate statistically differences ($p \leq 0.05$) between the different formulations under the same conditions of using or not using lipase.

[2] OPD green Extract refers to the green extracts obtained from *Opuntia stricta var. dillenii*'s whole fruits. [3] Formulation A, based on Tween 20 and Formulation B, based on caseinate.
